# Zulfiqar Frailty Scale (ZFS): Concordance Study with the Clinical Frailty Scale (CFS)

**DOI:** 10.3390/medicines9110058

**Published:** 2022-11-18

**Authors:** Abrar-Ahmad Zulfiqar, Léo Martin, Perla Habchi, Delwende Noaga Damien Massimbo, Ibrahima Amadou Dembele, Emmanuel Andres

**Affiliations:** 1 Service de Médecine Interne, Diabète et Maladies Métaboliques de la Clinique Médicale B, Hôpitaux Universitaires de Strasbourg et Equipe EA 3072 “Mitochondrie, Stress Oxydant et Protection Musculaire”, Faculté de Médecine-Université de Strasbourg, 67000 Strasbourg, France; 2 Anesthesiology Department, Aman Hospital, F Ring Rd, Zone 47, Building 412, Doha P.O. Box 8199, Qatar; 3 Service de Biostatistiques, CHRU Limoges, 87000 Limoges, France

**Keywords:** Zulfiqar Frailty Scale (ZFS), elderly subjects, Clinical Frailty Scale, prevention, primary care

## Abstract

Introduction: We designed a new scale for the rapid detection of frailty for use in primary care, referred to as the Zulfiqar Frailty Scale (ZFS). Objective: To evaluate the performance of the “ZFS” tool to screen for frailty as defined in the Clinical Frailty Scale (CFS) criteria in an ambulatory population of patients at least 75 years old. Method: A prospective study conducted in Alsace, France, for a duration of 6 months that included patients aged 75 and over was judged to be autonomous with an ADL (Activity of Daily Living) > 4/6. Results: In this ambulatory population of 124 patients with an average age of 79 years, the completion time for our scale was less than two minutes, and the staff required no training beforehand. Sensibility was 67%, while specificity was 87%. The positive predictive value was 80%, and the negative predictive value was 77%. The Youden index was 59.8%. In our study, we have a moderate correlation between CFS and ZFS (r = 0.674 with 95%CI = [0.565; 0.760]; *p*-value < 2.2 × 10^−16^ < 0.05). The Pearson correlations between these two geriatric scores were all strong and roughly equivalent to each other. The kappa of Cohen (k) = 0.46 (Unweighted), moderate concordance between the ZFS and CFS scales according to Fleiss classification. Conclusion: The “ZFS” tool makes it possible to screen for frailty with a high level of specificity and positive/negative predictive value.

## 1. Introduction

Targeting state of frailty is one of the priorities identified by the World Health Organization. As of 1 January 2021, more than one in five people (20.7%) in France were aged 65 or older. This percentage has been increasing for more than 30 years, and the aging of the population has accelerated since the mid-2010s—particularly with the aging of the first large generation born after WWII, the so-called “Baby Boomers.” The proportion of people aged 65 or over is increasing in every European Union (EU) country. In 2019, this group represented 20.0% of the EU population, compared to 17.4% in 2009 [1]. While there might not currently be an agreed-upon definition of “frailty syndrome,” there is, however, a reference scale that can be used: the Fried scale [2], taught in all universities and used daily in geriatric medicine departments. It is also called the “phenotypic fragility scale.” Developed in the 1990s by Professor Linda Fried’s (an American epidemiologist and geriatrician) teams, then adapted by the American Geriatric Society, it revolves around the concept of sarcopenia in phenotypic frailty [2]. However, this scale is not well suited to ambulatory medicine due to spatial constraints (walking over 4.57 m), the time needed, and material requirements (use of a dynamometer). Since then, using the multidimensional approach developed by the Canadian teams (Rockwood et al.) [3,4,5,6], many scales have emerged [7]—some of them remaining in an experimental stage. Their use in ambulatory medicine, in everyday practice, remains problematic. Some point to their time-consuming nature. Others believe these scales are difficult to adapt to general medicine practices, requiring specific equipment, such as a dynamometer or, moreover, medical consultation premises that allow walks of a given duration and distance to realize their full usefulness. As the elderly population grows in number and wants to stay at home as long as possible, it is essential to be able to detect a state of fragility in order to take all possible measures to reverse it. In addition, it should be noted that with the number of general practitioners decreasing, it is important to offer quick and clear scales, without any real training and at hand for the medical profession and also for other health professionals. It should be noted that geriatricians, at the international level by extension, have been unable to agree on a precise definition, which likely contributes to the multiplicity of current frailty scales. One of the grievances also mentioned is the lack of consultation with general practitioners (GPs) in the creation and implementation of frailty scales. A scale created in a hospital without consulting general practitioners can be difficult to accept because it does not necessarily integrate all the constraints primary care medical practices face. Therefore, it is essential to promote this partnership between general medicine and hospitals and generally between different medical specialties and geriatrics. This partnership is even more critical since the general practitioners are the doctors who will see most elderly subjects as outpatients in their clinics or on occasional home visits. It is in this context that we have proposed a new rapid screening scale for frailty (known as “ZULFIQAR”), designed to be used by general practitioners [8]. We tested and validated it at other general medicine practices to see how easy it is to reproduce. We evaluated its performance and compared it with the reference frailty scale, the Fried scale, the GFST scale, and the SEGA scale, with very satisfactory results [8]. Our frailty screening scale has been the subject of several published (or soon-to-be-published) studies since the original article appeared in the MEDICINES MDPI journal. The proof-of-concept study results were very satisfactory and reproducible, and similar results have been found in subsequent trials [8,9,10].

The Clinical Frailty Scale is a clinical judgment-based frailty tool developed for the Canadian Study of Health and Aging, which originated from Dalhousie University in Canada [3,11]. Based on the biological and physiological criteria of frailty, this scale emphasizes functionality. The description of frailty levels is based on simple clinical observations, including comorbidities, functional status, and activity level. The rating is based on the judgment of the administrator [7]. The objective of this preliminary task is to determine the performance of the “Zulfiqar Frailty Scale (ZFS)” tool to detect frailty (defined by the CFS) in this ambulatory population.

## 2. Patients and Methods

### 2.1. Methods

Prospective study was conducted at four general medicine practices in the Alsace region, specifically in Neuf Brisach, Saint-Louis, Rixheim, and Sarre-Union, for a total period of 9 months (from 17 May 2021 to 10 February 2022).

### 2.2. Primary Objective

The objective of the study was to validate the Zulfiqar Frailty Scale (ZFS) and to analyze its concordance with Clinical Frailty Scale (CFS) as established by Rockwood et al. and translated into French [3,12].

### 2.3. Inclusion Criteria

The patients needed to be 75 years of age or over, in consultation with a general practitioner, and with an ADL (Activity of Daily Living) greater than or equal to four.

### 2.4. Exclusion Criteria

Patients less than 65 years old and subjects with an ADL of less than four were excluded from this study. Those living in nursing homes were also excluded, as were patients unable to express themselves or give their consent.

### 2.5. Data Collected and Analyzed

Data for the study was recorded by the general practitioner during routine consultations. The Clinical Frailty Scale, as well as the Zulfiqar Frailty Scale, was screened for each patient. The information was then anonymized before being transmitted for collection in the study.

#### 2.5.1. Frailty Screening with the “Zulfiqar Frailty Scale” (ZFS) Tool

The score has six items. A point was assigned for each positive indicator (maximum score = 6). See details about the ZFS tool in Table 1.

#### 2.5.2. Frailty Screening with the “Clinical Frailty Scale” (CFS)

We used the Clinical Frailty Scale (CFS) in our study, evolving from the Canadian Study of Health and Aging. It was developed as a grading tool with seven scales in 2005 [3] and revised in 2008 to a 9-point scale [13]: 1-Very Fit; 2-Well; 3-Managing Well; 4-Living With Very Mild Frailty; 5-Living with Mild Frailty; 6-Living With Moderate Frailty; 7-Living With Severe Frailty; 8-Living with Very Severe Frailty; 9-Terminally Ill. For scores of 5 or more, the elderly patient was considered by CFS to be “frail”.

### 2.6. Statistical Analysis

The data was analyzed by XL-Stat software (Addinsoft Inc., New York, NY., USA) and collected anonymously using an MS Excel spreadsheet. First, a descriptive analysis of the results obtained was prepared. Quantitative variables were expressed as mean ± standard deviation and qualitative variables as absolute and relative numbers (percentages). Next, the “ZFS” tool was compared to CFS using the student’s *t*-test for quantitative variables and Chi² for qualitative variables. It was used to measure the performance of a diagnostic test and to determine optimal threshold values. The relative risk of each item in the “ZFS” tool was estimated by calculating the Odds ratio, with a 95% confidence interval defined by the Miettinen method, resulting in a 5% alpha probability. The “ZFS” score was assessed in terms of sensitivity, specificity, Youden’s index, positive and negative predictive values, and the area under the ROC curve, using the CFS score as the gold standard. A Kappa coefficient and Spearman coefficient were calculated to measure the concordance between the two tests. A Pearson correlation matrix was used to evaluate discrepancies between the total scores and the items of each score.

### 2.7. Administrative Elements

The work conforms to the provisions of the Declaration of Helsinki (as revised in Tokyo 2004). The material contained in the manuscript has not been previously published and is not being concurrently submitted elsewhere. Informed consent was obtained from all patients included in this study. From a regulatory standpoint, the paper has received ethical approval and is registered with the Comité de Protection des Personnes, registration number: 2022-A01817-36.

## 3. Results

### 3.1. Description of the Population

**Figure 1:**
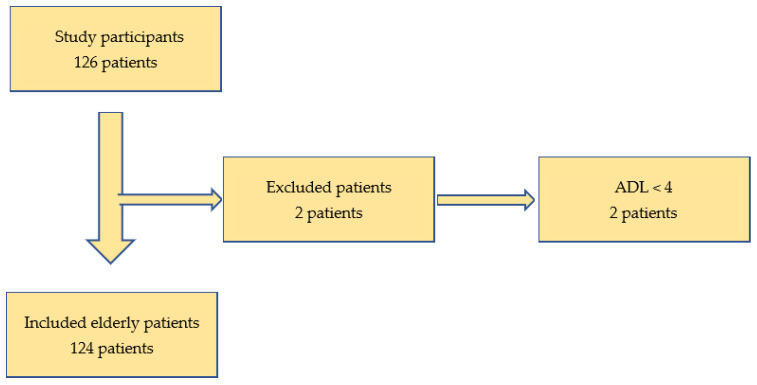
Flowchart. ADL: Activity of Daily Living.

During this collection period, 124 patients over 75 years of age were included (see Figure 1); the group was comprised of 64 women (52%) and 60 men (48%). We did not note any refusals. The characteristics of the population included are detailed in Table 2. Table 3 specifies the characteristics of the frailty scales used (the frailty scale known as Zulfiqar Fraily Scale (ZFS) and the Clinical Frailty Scale (CFS)).

### 3.2. Performance and Validity of the Zulfiqar Frailty Scale

A comparison of the element scores of the Zulfiqar Frailty Scale between frail and non-frail patients was made (see Table 4). All results evaluating our screening tool against the CFS are shown in Table 5.

Table 5 displays the full results of our screening tool using CFS’s criteria.

### 3.3. Correlation between the CFS and Zulfiqar Frailty Scale

Pearson’s correlation coefficient (or Pearson’s r) and its 95% confidence interval was 0.674 (0.56; 0.76) (*p* < 0.001). In our study, there was a moderate correlation between the “ZFS” and the CFS/9.

The following matrix presents the Phi coefficients (equivalent to Pearson correlation coefficients) between items. The correlation between the ZFS items and general and geriatric indicators were weak. See heatmap below (Table 6)

Spearman’s correlation coefficient (called Rho) between the ZFS and CFS scores is 0.69 with a *p*-value < 2.2 × 10^−16^. The kappa of Cohen (k) = 0.46 (Unweighted), moderate concordance between the ZFS and CFS scales according to Fleiss classification (*p* < 1.56 × 10^−0.8^).

See Table 7 for the contingency study.

**Figure 2:**
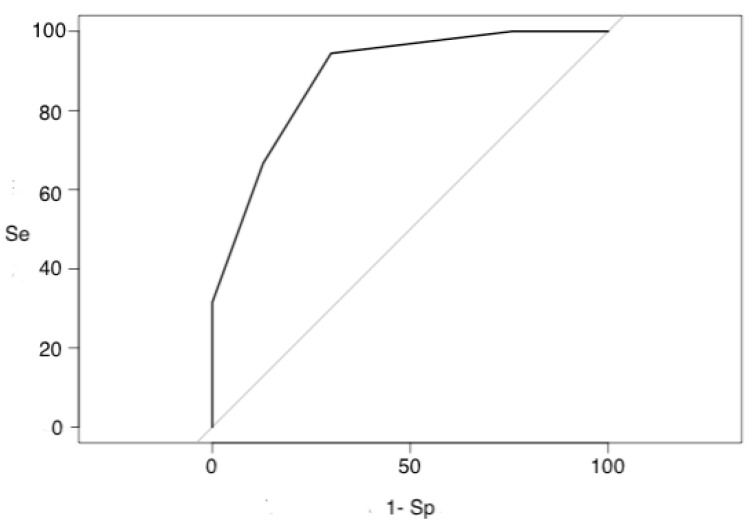
ROC curve ZFS.

Finally, the area under the curve (ROC) of the Zulfiqar Frailty Scale was 0.88 (0.83; 0.94) (IC95%). See Figure 2 and Table 8.

## 4. Discussion

We developed a frailty screening tool that standardizes professional procedures and makes it possible for general practitioners to detect frailty in their elderly patients. The results of this study are very satisfactory and similar to previous studies [8,9,10]: in fact, the correlations between the Zulfiqar scale (and the simplified scale) and other frailty scales are very satisfactory [14,15,16,17,18]. In addition, the areas under the curve ranged from 0.70 to 0.94. These results show that the ZFS and sZFS are outstanding tools for detecting frailty. Our goal was to create a rapid frailty screening scale that would be useful for general practitioners. Our scale aims to facilitate the early detection of frail elderly people, which will help to delay the loss of their autonomy. Our tool does not require any equipment whatsoever, making it advantageous and perfectly suitable for primary care. The screening is quick and easy, allowing physicians to do away with other time-consuming methods that inconvenience elderly patients.

The CFS has been validated as an adverse outcome predictor in hospitalized older people [19,20,21].

This frailty scale is used mainly in hospitals, for hospitalized patients [21], particularly in emergency rooms and intensive care/resuscitation units [21,22]—and, especially during the COVID-19 pandemic [23,24,25], it plays a supporting role in triage. It has also been used for hospitalized patients in cardiology [26,27], orthopedics [28], geriatrics [29,30], and other units [31]. Less has been published about its use in general medicine than its use in hospitals [21]. It requires professional training to ensure the practitioner knows how to use it and understands geriatric syndromes. The practitioner must use their judgment. It is also important to note this scale, rated out of 9, goes beyond frailty syndrome: items 7, 8, and 9 are more characteristic of dependency than frailty syndrome. In addition, there is very little difference between items 5 and 6—which is why detailed training for medical and paramedical professionals is needed. Our scale can quickly test all the main frailty syndromes, covering nutrition, socialization, iatrogenic issues, memory, and falls. The questions are simple, based on information available to any health professional, with no prior training necessary. It can be used by general practitioners on a daily basis but can also be administered by nurses, physiotherapists, occupational therapists, or even social workers.

The ZFS scale has the advantage of being composed of six objective items, covering the geriatric syndrome as a whole through nutrition, socialization, memory, falls, and iatrogenics. The items are simple, with a binary answer (yes or no), based on information accessible to any health professional and requiring no prior training. It can be used by general practitioners, nurses, physiotherapists, occupational therapists, or social workers, and this is without any variability between examiners. The ZFS scale is a rapid screening scale for frailty syndrome dedicated to general medicine and ambulatory medicine/primary care. Many scales, both on the phenotypic and multidimensional levels, have emerged. However, due to a mismatch between some of these scales and ambulatory medicine (linked to the time-consuming nature, unsuitability to practices, and a certain level of knowledge to be acquired), these scales remain very little or not used in general medicine. In addition, most scales have been designed and developed in a hospital setting, which is the case with the Clinical Frailty Scale (CFS). In contrast, the ZFS scale has been designed by general medicine and applied only in outpatient medicine.

Our scale can be used in less than two minutes, concurring with our own data—which increases its usefulness in outpatient care. The general practitioner has all the patient’s information about iatrogenic issues, level of social activity, and nutrition. Each item in the questionnaire is scored a “1” if present, for a maximum total of “6”.

The tool is easy to use in a general medical consultation; therefore, it is appropriate for use in multidisciplinary nursing homes where a frailty diagnosis could be accompanied by specific follow-up measures, thanks to the variety of professionals present. Due to the lack of many “frailty day hospitals” and the shortage of geriatricians, the tool could be a significant add-on. The general practitioner can, of course, be the one to initiate the personalized care plan and play a central role in it. The doctor will coordinate care with paramedical and social stakeholders, reassess patient care, and guide family caregivers. In practice, 30–40% of seniors living at home suffer from frailty.

Given the ubiquity of this syndrome and the existence of medical deserts, screening for frailty relies on local paramedical professionals (nurses, physiotherapists, pharmacists, etc.). Therefore, we have also launched a dedicated website, accessible everywhere—in cities, suburban, and rural areas, for all medical professionals, and also for paramedical professionals, such as at-home nurses, physiotherapists, and occupational therapists: http://zulfiqarfrailtyscale.com/ (accessed on 15 August 2022).

Our work is only limited by its monocentric character and the small sample size. A prospective study conducted across several sites with multiple practitioners would have made it possible to better understand the scope of administrator judgment needed regarding the CFS scale. The predictive nature of our scale, as it pertains to undesired events, such as hospitalizations or falls, has not been studied; however, this will be the subject of further research and investigation.

## 5. Conclusions

The Zulfiqar Frailty Scale seems to be suitable for wide use in primary care. The objective of our scale is to provide the ability to conduct rapid screening for frailty syndrome on an outpatient basis. By offering general practitioners a simple and straightforward tool for rapid screening during routine medical consultations, we enable them to refer frail patients to a gerontological team for further evaluation. The next stop is to study the ability to predict potentially dangerous situations, this will take place in the upcoming weeks and months.

## Figures and Tables

**Table 1:** Zulfiqar Frailty Scale (ZFS).

Weight Loss Greater than or Equal to 5% in 6 Months?	Yes	No
Monopod support test < 5 s?	Yes	No
Living alone?	Yes	No
Home caregivers?	Yes	No
Memory loss?	Yes	No
More than 5 therapeutic classes on his/her prescription history for less than 6 months?	Yes	No

Score of 0: non-frail. Scores of 1 or 2: pre-frail. Scores of 3 or more: frail.

**Table 2:** Description of the sample.

	Mean (sd)	Median [Q25–75]	Min	Max	
Age	79.1 (3.60)	79.0 (76.0; 81.0)	75.0	91.0	124
Weight kg	75.4 (15.9)	74.0 (63.0; 88.2)	44.0	119.0	124
BMI kg/m	26.6 (5.29)	25.7 (22.8; 30.0)	17.4	43.0	124
ADL/6	5.65 (0.573)	6.00 (5.50; 6.00)	4.00	6.00	124
IADL/8	7.40 (1.01)	8.00 (7.00; 8.00)	4.00	8.00	124
Charlson	5.67 (1.77)	5.00 (4.00; 7.00)	3.00	10.0	124
Medication	5.30 (2.31)	5.00 (4.00; 7.00)	0	13.0	124

BMI: Body Mass Index; ADL: Activity Daily Living; IADL: Instrumental Activity Daily Living.

**Table 3:** Zulfiqar Frailty Scale (ZFS) and Clinical Frailty Scale (CFS).

Zulfiqar Frailty Scale (ZFS)				
			*n* = 124	
Zulfiqar Frailty Scale (ZFS)				
			*n* (%)	Mean (sd)
Weight loss?			17 (14%)	/
Monopod support test < 5 s?			64 (52%)	/
Living alone?			37 (30%)	/
Home caregivers?			30 (24%)	/
Memory loss?			29 (23%)	/
More than 5 therapeutic classes?			76 (61%)	/
	ZFS score		/	2 (1.5)
	Duration time, m		/	141.2 (22.8)
	Frailty (ZFS > = 3/6)		45 (36%)	
CFS score				3.4 (1.2)
	Frailty according to CFS (score CFS > 4)		25 (20%)	

**Table 4:** Presents a comparison of the element scores of the Zulfiqar Frailty Scale between frail and non-frail patients.

Zulfiqar Frailty Scale (ZFS)				
	Frail, *n* = 45	Non-Frail, *n* = 79	*p*-Value	Significativity
Weight loss?			1.57 × 10^−4^	***
Yes	13 (29%)	4 (5%)		
No	32 (71%)	75 (95%)		
Monopod support test < 5 s?			1.01 × 10^−11^	****
Yes	40 (89%)	24 (30%)		
No	5 (11%)	55 (70%)		
Living alone?			1.80 × 10^−3^	**
Yes	21 (47%)	16 (20%)		
No	24 (53%)	63 (80%)		
Home caregivers?			3.97 × 10^−15^	****
Yes	27 (60%)	3 (4%)		
No	18 (40%)	76 (96%)		
Memory loss?			1.64 × 10^−6^	****
Yes	21 (47%)	8 (10%)		
No	24 (53%)	71 (90%)		
More than 5 therapeutic classes?			6.58 × 10^−8^	****
Yes	41 (91%)	35 (44%)		
No	4 (9%)	44 (56%)		

**** < 0.0001; ***: < 0.001; **: < 0.01.

**Table 5:** Summary of all ZFS tool results compared to CFS.

(CFS)								
	Frail, *n* = 25	Not Frail, *n* = 99	*p*-Value	Se	Sp	PPV	NPV	Youden Index
Weight?			0.31					
Yes	5 (20%)	12 (12%)		20%	88%	29%	90%	8%
No	20 (80%)	87 (88%)						
Monopod support test < 5 s?			2.87 × 10^−6^					
Yes	23 (92%)	41 (41%)		92%	59%	36%	97%	51%
No	2 (8%)	58 (59%)						
Living alone?			0.794					
Yes	8 (32%)	29 (29%)		32%	70%	22%	80%	2%
No	17 (68%)	70 (70%)						
Home caregivers?			1.23 × 10^−11^					
Yes	18 (72%)	12 (12%)		72%	88%	60%	93%	60%
No	7 (28%)	87 (88%)						
Memory problems?			0.006					
Yes	11 (44%)	18 (18%)		44%	81%	38%	85%	25%
No	14 (56%)	81 (81%)						
More than 5 therapeutic classes on his/her prescription history for less than 6 months?			3.37 × 10^−4^					
Yes	23 (92%)	53 (54%)		92%	46%	29%	96%	38%
No	2 (8%)	46 (46%)						

Se: sensibility; Sp: specificity; PPV: positive predictive value; NPV: negative predictive value.

**Table 6:** Correlation matrix.

	Age	Gender	Weight (kg)	Height (cm)	BMI (kg/m)	Charlson Score	Hospitalization < 6 Months	Medication	Fall < 6 Months
Weight loss	0.02	0.08	−0.23	0.01	−0.24	0.09	0.13	0.11	−0.11
Risk of fall	0.21	0.03	0.23	0.01	0.23	0.34	0.24	0.31	0.25
Home alone	0.24	−0.07	−0.19	−0.06	−0.17	0.06	0.00	0.04	0.05
Caregivers	0.20	−0.02	−0.03	−0.05	−0.01	0.43	0.30	0.36	0.26
Memory loss	0.24	−0.04	−0.06	0.16	−0.08	0.24	0.17	0.29	0.12
Medication	0.10	0.07	0.08	0.01	0.10	0.44	0.26	0.76	0.03

BMI: Body Mass Index.

**Table 7:** Contingency table—Zulfiqar Frailty Scale vs. CFS.

	CFS Frail	CFS Not Frail
ZFS frail	36	9
ZFS not frail	18	61

**Table 8:** ROC curve interpretation thresholds.

Threshold	Se	Sp	1-Sp	Youden	PPV	NPV
0	1	0	1	0%	44%	/
1	1	0.24	0.76	19%	49%	85%
2	0.94	0.70	0.30	64%	71%	94%
3	0.66	0.87	0.13	54%	80%	77%
4	0.31	1	0	31%	100%	65%
5	0.18	1	0	19%	100%	61%
6	0.04	1	0	4%	100%	57%

## Data Availability

The datasets used and/or analyzed during the current study are available from the corresponding author on reasonable request.
